# Conversion From Calcineurin Inhibitors to Mammalian Target of Rapamycin Inhibitors in Kidney Transplant Recipients: A Systematic Review and Meta-Analysis of Randomized Controlled Trials

**DOI:** 10.3389/fimmu.2021.663602

**Published:** 2021-09-03

**Authors:** Jun Zeng, Qiang Zhong, Xiaobing Feng, Linde Li, Shijian Feng, Yu Fan, Turun Song, Zhongli Huang, Xianding Wang, Tao Lin

**Affiliations:** ^1^Department of Urology, Institute of Urology, West China Hospital, Sichuan University, Chengdu, China; ^2^Organ Transplantation Center, West China Hospital, Sichuan University, Chengdu, China

**Keywords:** calcineurin inhibitor, mammalian-target-of-rapamycin inhibitor, kidney transplantation, conversion, meta-analysis

## Abstract

**Background:**

A systematic review and meta-analysis were performed to investigate the efficacy and safety of conversion from calcineurin inhibitors (CNIs) to mammalian target of rapamycin inhibitors (mTORi) in kidney transplant recipients (KTRs).

**Methods:**

MEDLINE, EMBASE, PubMed, and Cochrane Library were searched to identify randomized controlled trials (RCTs) that compared the continuation of CNI with conversion to mTORi therapy.

**Results:**

Twenty-nine RCTs (5,747 KTRs) were included in our analysis. Meta-analysis of the glomerular filtration rate (SMD 0.20; 95%CI 0.10–0.31; P<0.01) and malignancy (RR 0.74; 95%CI 0.55–0.99; P=0.04) demonstrated a significant advantage of mTORi conversion over CNI continuation. However, the risk of acute rejection (RR 1.58; 95%CI 1.22–2.04; P<0.01), infection (RR 1.55; 95%CI 1.01–1.31; P=0.04), proteinuria (RR 1.87; 95%CI 1.34–2.59; P<0.01), leukopenia (RR 1.56; 95%CI 1.27–1.91; P<0.01), acne (RR 6.43; 95%CI 3.43–12.04; P<0.01), and mouth ulcer (RR 11.70; 95%CI 6.18–22.17; P<0.01) were higher in the mTORi group. More patients in the conversion group had to discontinue study medication (RR 2.52; 95%CI 1.75–3.63; P<0.01). There was no significant difference between the two groups with regard to death, graft loss, diabetes, chronic allograft nephropathy, and interstitial fibrosis/tubular atrophy.

**Conclusions:**

Posttransplant patients have a better graft function and lower incidence of malignancy after conversion from CNI to mTORi therapy. However, this conversion strategy may be prevented by the higher drug discontinuation rate due to mTORi-associated adverse events, such as more acute rejection, infection, proteinuria, leukopenia, acne, and mouth ulcer, indicating that conversion therapy may only be a treatment option in selected patients.

## Introduction

Most kidney transplant recipients (KTRs) currently receive calcineurin inhibitor (CNI) therapy, which has remarkably reduced acute rejection (AR) episodes and improved early graft survival (1). However, long-term CNI exposure may induce irreversible nephrotoxicity, resulting in progressive graft dysfunction (2, 3). The CNI can also promote cardiovascular events and malignancies, which are the leading causes of premature death with a functioning graft (4, 5). This discrepancy has prompted investigations into CNI retention strategies, which maintain adequate immunosuppressive effects without compromising safety (6).

Mammalian target of rapamycin inhibitors (mTORi) are new immunosuppressants that exhibit little or minimal nephrotoxicity (7–9). The dual immunosuppressive and antineoplastic properties of mTORi offer a distinct advantage for this class of drugs in the treatment of patients who receive kidney transplant (10, 11). Nevertheless, major concerns associated with the *de novo* introduction of mTORi include the risk of AR, impaired wound healing, and prolonged delayed graft function (DGF), limiting the adoption of mTORi as a first-line immunosuppressant (9, 12, 13).

Thus, increasing interest has been directed toward exploiting the synergistic actions of CNIs and mTORi by administering the CNI at the initial stage of high-risk AR after transplantation and then converting it into mTORi before the onset of CNI-induced irreversible nephrotoxicity (14). Evidence regarding the clinical benefit of this conversion strategy is conflicting. Budde et al. (15) demonstrated that mTORi combined with CNI withdrawal improved kidney function while maintaining efficacy and safety. In contrast, several studies reported that mTORi therapy not only did fail to show any overall clinical benefit but also mTORi conversion was associated with more adverse events (16) and discontinuations (17). Thus, further evidence for the validity of mTORi conversion strategy is urgently needed (18). Our study aimed to systematically identify and evaluate the currently available evidence from randomized controlled trials (RCTs) on the outcomes of conversion from the CNI to mTORi for KTRs.

## Materials and Methods

Our systematic review was conducted and reported in accordance with the Preferred Reporting Items for Systematic Reviews and Meta-Analyses (PRISMA) guidelines (19).

### Eligibility Criteria

Studies were eligible if they met the following criteria: (a) RCTs of conversion from CNIs to mTORi maintenance immunosuppressive regimen; (b) participants were the recipients of a single organ kidney transplant (first or repeat) from a living or deceased donor; (c) initial immunosuppression consisted of a CNI but not an mTORi; and (d) KTRs were randomly assigned to either continue with current CNI therapy or be converted from the CNI to mTORi. Dose modifications of CNIs, mTORi, and other concomitant immunosuppressants were permissible after randomization. Randomized trials with less than 20 cases in each group or abstracts of major transplant conferences were excluded. KTRs with a history of malignancy (other than adequately treated non-melanoma skin carcinoma) before randomization were excluded. To minimize participant overlap, when the same study was reported many times, the study with long follow-up period and complete case report was identified as the primary data source.

### Search Strategy

MEDLINE, EMBASE, PubMed, and Cochrane Library were searched from the start date of each resource up to November 12, 2020 (the last literature search) using logical combinations of relevant keywords and medical subject headings that contained all spellings of the following terms: kidney transplantation, calcineurin inhibitor, CNIs, tacrolimus, cyclosporine, conversion, mTORi, sirolimus, and everolimus. No restrictions regarding the language of the publication, age of KTRs, or concomitant immunosuppressants were applied. The results were supplemented by manually searching reference lists of relevant reviews and included articles. All citations identified by this search strategy were evaluated by two independent reviewers (JZ and QZ) using titles, abstracts, and, where required, the full text to determine eligibility.

### Outcome Measures

The primary endpoints were graft function, AR, graft loss, and death. The secondary outcomes were adverse events (AEs), infections, malignancies, and discontinuation of medication.

### Data Extraction

Eligible RCTs were referred to throughout this paper by the first author (JZ and QZ) and the year of the earliest peer-reviewed publication. Data extraction was independently performed by two reviewers (JZ and QZ) using a predesigned form on which study design, participant characteristics, interventions, and outcome data were recorded. Missing information was requested from the trial authors or sponsors. They met to combine their findings, and the information was subsequently entered into Review Manager (RevMan) (Version 5.4, Copenhagen: The Nordic Cochrane Centre, The Cochrane Collaboration).

### Quality Assessment and Statistical Analyses

Cochrane Collaboration’s tool was used to assess the risk of bias of randomized controlled trials (20). RevMan 5.4 was utilized for the execution of the meta-analysis. Dichotomous outcomes were expressed as risk ratios (RRs) with 95% confidence intervals (CIs), and continuous variables were expressed as mean differences (MDs) with 95% CI. Data were pooled using the standardized mean difference (SMD) as the summary effect size metric. P<0.05 was considered statistically significant. Meta-analysis was conducted using the fixed effect model in the absence of heterogeneity; otherwise, the random effect was applied. Heterogeneity was quantified *via* the Cochrane Q (P<0.1) and I^2^ statistics (I^2^>50%). If heterogeneity existed, a sensitivity analysis was performed to explore possible sources of heterogeneity.

## Results

### Search and Selection

**Figure 1:**
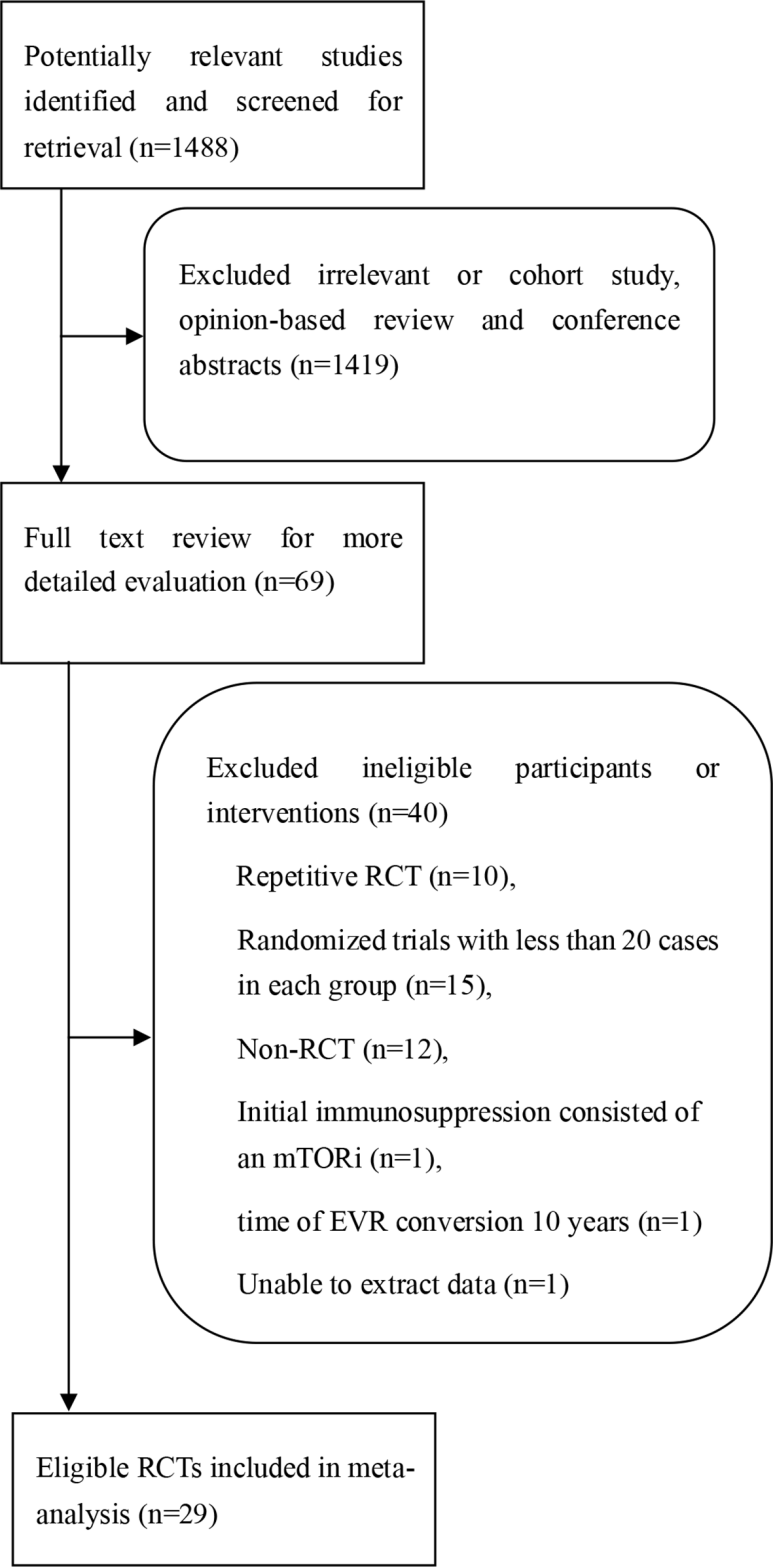
Procedure for the search and selection of RCTs included in the systematic review and meta-analysis.

The search and selection flow chart is illustrated in **Figure 1**. Initial literature searches identified 1,488 citations across all databases. The titles and abstracts were reviewed, followed by full-text review of potentially eligible articles and abstracts, of which 29 RCTs met the inclusion criteria (15, 17, 21–47). The baseline overview of included studies is reported in **Table 1**.

**Table 1:** Baseline overview of 29 included studies.

Study	Year	Design	Sample size	Time since transplantation	Follow-up (months)	Results				
						eGFR	Death	Graft loss	AR	AEs
Tönshoff	2019	RCT	52/54	4–6 weeks	12	Yes	No	No	Yes	Yes
Taber	2019	RCT	30/30	3 months	12	Yes	No	No	Yes	Yes
Brakemeier	2019	RCT	51/24	7 weeks	6	Yes	No	No	Yes	Yes
Haynes	2018	RCT	197/197	6 months	18	Yes	Yes	Yes	Yes	Yes
Bouamar	2018	RCT	30/30	3 months	12	Yes	Yes	Yes	Yes	Yes
Fijter	2017	RCT	359/356	10–14 weeks	24	Yes	Yes	Yes	Yes	Yes
Budde	2017	RCT	171/165	3 months	12	Yes	Yes	Yes	Yes	Yes
Bemelman	2017	RCT	96/89	6 months	24	NR	Yes	Yes	Yes	Yes
Pretagostini	2016	RCT	24/25	1-month	12	NR	NR	NR	Yes	Yes
Felix	2016	RCT	60/59	3 months	24	NR	Yes	NR	Yes	Yes
Cruzado	2016	RCT	35/36	6 months to 3 years	24	Yes	Yes	No	No	Yes
Rostaing	2015	RCT	96/98	3–4 months	12	Yes	No	Yes	Yes	Yes
Mjörnstedt	2015	RCT	92/90	7 weeks	24	Yes	Yes	No	Yes	Yes
Sandes-Freitas	2015	RCT	48/45	3 months	24	Yes	NR	NR	Yes	NR
Budde	2015	RCT	46/47	NR	12	Yes	Yes	No	No	Yes
Silva	2013	RCT	97/107	3 months	24	Yes	Yes	Yes	Yes	Yes
Chhabra	2013	RCT	123/64	12 months	24	Yes	Yes	Yes	Yes	Yes
Bansal	2013	RCT	31/29	2 months	6	Yes	NR	NR	Yes	Yes
Mjörnstedt	2012	RCT	102/100	7 weeks	12	Yes	Yes	No	Yes	Yes
Weir	2011	RCT	148/151	1–6 months	24	Yes	Yes	Yes	Yes	Yes
Holdaas	2011	RCT	127/123	6 months	24	Yes	Yes	Yes	Yes	Yes
Heilman	2011	RCT	62/60	1-month	24	Yes	Yes	Yes	Yes	Yes
Budde	2011	RCT	154/146	4.5 months	12	Yes	Yes	No	Yes	Yes
Guba	2010	RCT	69/71	10–24 days	12	Yes	Yes	Yes	Yes	Yes
Schena	2009	RCT	555/275	6–120 months	24	Yes	NR	Yes	Yes	Yes
Lebranchu	2009	RCT	95/97	3 months	12	Yes	No	Yes	Yes	Yes
Bemelman	2009	RCT	38/39	6 months	24	Yes	NR	NR	Yes	Yes
Liu	2007	RCT	56/54	> 12 months	24	NR	NR	No	No	NR
Stallone	2005	RCT	34/50	12–36 months	24	NR	NR	NR	No	Yes

eGFR, estimated glomerular filtration rate; AR, acute rejection; AEs, adverse events; NR, not reported.

### Description of Eligible Trials

There were 29 eligible RCTs, in which a total number of 5,747 KTRs were included, whose characteristics are summarized in **Table S1**. Among these 29 trials, 22 trials reported the time of conversion to mTORi within 6 months after transplantation; the rest were more than 6 months. A total of 16 RCTs reported the impressive therapy of conversion from the CNI to everolimus. The patient sample size ranged from 49 to 830, and follow-up duration was from 6 to 24 months.

### Quality of Included Trials

The quality of the 29 clinical trials was evaluated by Cochrane Collaboration’s tool (**Figure S1**). A total of 14 studies exhibited an adequate random sequence generation process, 15 trials described the methods used for allocation concealment, 2 RCTs illustrated performance bias, and 4 studies had unreported data. In short, the studies included had low to moderate risk of bias.

### Primary Endpoints

**Figure 2:**
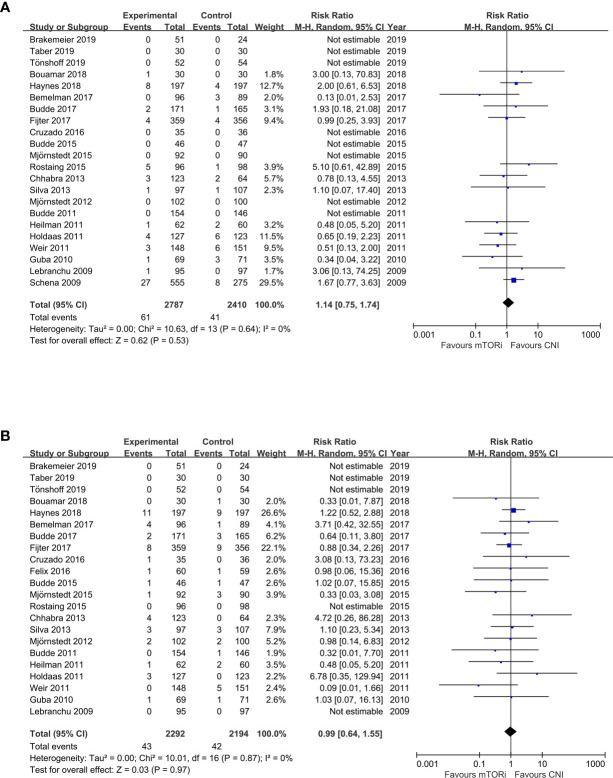
The forest plot of studies that compared conversion from CNIs to mTOR inhibitors versus maintenance of CNI therapy for the outcomes of **(A)** renal function (eGFR) and **(B)** acute rejection (AR).

**Figure 3:**
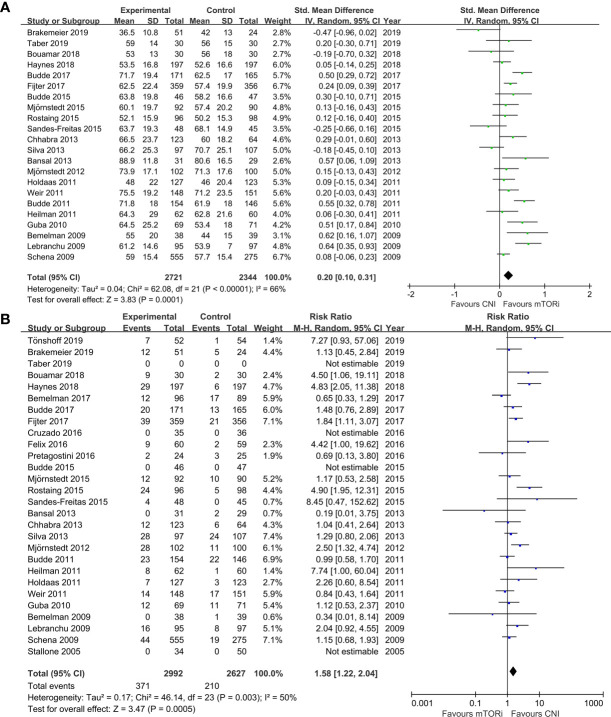
The forest plot of studies that compared conversion from CNIs to mTOR inhibitors versus maintenance of CNI therapy for the outcomes of **(A)** graft loss and **(B)** mortality.

The meta-analysis results of the primary endpoints are reported in **Figures 2** and **3**. A total of 22 trials reported renal function; the meta-analysis of the estimated glomerular filtration rate (eGFR) demonstrated a statistically significant advantage for mTORi conversion over CNI continuation (SMD 0.20; 95%CI 0.10–0.31; P<0.01) in **Figure 2A**. A total of 28 trials reported AR; we found that patients converted to mTORi were at a higher risk of developing AR (RR 1.58; 95%CI 1.22–2.04; P<0.01) in **Figure 2B**. A total of 22 trials reported graft loss; there was no difference between patients converted to mTORi and those remaining on the CNI (RR 1.14; 95%CI 0.75–1.74; P=0.53) in **Figure 3A**. A total of 22 trials reported mortality; we also found that there was no statistical difference in mortality between the mTORi conversion group and the CNI continuation arm (RR 0.99, 95%CI 0.64–1.55; P=0.97) in **Figure 3B**.

### Secondary Endpoints

Sixteen studies described the adverse events (AEs) and serious adverse events (sAEs). Despite the comparable rate of AEs (RR 1.03; 95%CI 0.99–1.06; P=0.11), patients converted into mTORi had a significantly higher risk to have sAEs (RR 1.23; 95%CI 1.09–1.39; P<0.01; **Figure S2A**). Twenty-one RCTs reported the drug discontinuation from AEs. There was a greater risk of drug discontinuation in patients who converted to mTORi compared with those on the CNI (RR 2.52; 95%CI 1.75–3.63; P<0.01; **Figure S2B**). Sixteen trials described infections, and eight trials described serious infections (defined as requiring hospitalization). We found that there was a higher risk of infections and serious infections in the mTORi conversion group compared with the remaining CNI group (RR 1.15; 95%CI 1.01–1.31; P=0.04; **Figure S3A**; RR 1.17; 95%CI 1.05–1.30; P<0.01; **Figure S3B**, respectively). Fifteen studies reported on cancer outcomes. Patients converted from the CNI to mTORi had lower risk of malignancy compared with those continuing on the CNI therapy (RR 0.74; 95%CI 0.55–0.99; P=0.04; **Figure S4A**). In addition to these advantageous results, we also found other adverse consequences brought about by the mTORi therapy, including proteinuria (RR 1.87; 95%CI 1.34–2.59; P<0.01; **Figure S4B**), mouth ulcer (RR 11.70; 95%CI 6.18–22.17; P<0.01; **Figure S4C**), anemia (RR 1.55; 95%CI 1.26–1.89; P<0.01; **Figure S5A**), leukopenia (RR 1.56; 95%CI 1.27–1.91; P<0.01; **Figure S5B**), thrombocytopenia (RR 2.45; 95%CI 1.13–5.35; P=0.02; **Figure S5C**), dyslipidemia (RR 1.41; 95%CI 1.27–1.58; P<0.01; **Figure S6A**), acne (RR 6.43; 95%CI 3.43–12.04; P<0.01; **Figure S6B**), diarrhea (RR 1.46; 95%CI 1.28–1.67; P<0.01, **Figure S7A**), and edema (RR 1.49; 95%CI 1.14–1.93; P<0.01; **Figure S7B**). In **Figure S8**, there were no differences in diabetes and interstitial fibrosis/tubular atrophy (IF/TA) between the mTORi conversion group and the CNI continuation group. On the other hand, there was no significant difference between the two groups with regard to CMV or BKV infection (**Figure S9**), chronic allograft nephropathy (increased serum creatine), gastroenteritis, pyrexia, posttransplant lymphoproliferative disease (PTLD), and wound-related problem (**Table S3**).

## Discussion

The meta-analysis of the 29 eligible RCTs (involving 5,747 KTRs) demonstrated that the conversion from the CNI to mTORi after kidney transplantation was associated with an improvement in graft function and a reduced incidence of malignancy. There was no significant difference between the groups with respect to IF/TA or chronic allograft nephropathy, indicating that the improvement in graft function after conversion may primarily be due to effects other than structural improvement (48). Ten years ago, mTOR inhibitors were more commonly used, but only 1.9% of recipients were prescribed with them at transplant in 2016, increasing to 4.3% at 1-year posttransplant (49). Current studies reported that the combination of low-dose CNI (tacrolimus) and everolimus can make the difference in order to obtain the best clinic benefit for KTRs (50). Nevertheless, conversion to mTORi therapy may be prevented by the high discontinuation rate (51) due to the intolerable AEs, such as the development of serious infections, AR, mouth ulceration, proteinuria, anemia, leucopenia, thrombocytopenia, dyslipidemia, acne, diarrhea, and edema. All of these changes were statistically significant between the two groups. Thus, the application of mTORi conversion must be weighed during clinical decision-making, with the potential benefits and risks carefully assessed for each KTR.

Previous systematic review had suggested that conversion to sirolimus was associated with an improvement in short-term renal function, while none of the identified studies investigated everolimus (18, 52). Similarly, Lim et al. (53) reviewed RCTs comparing delayed conversion from CNIs to mTORi indicating that patients converted to mTORi up to 1-year posttransplant had higher GFR compared with those remaining on the CNI. Those findings were highly consistent with our meta-analysis, but we also found that there was no difference in CMV and BKV infection, and no difference in new-onset diabetes. Wolf et al. (54) concluded that the incidence of infections is lower when mTORi is combined with a CNI compared to a standard CNI therapy following renal transplantation, while we found that mTORi conversion therapy had more risk of infections.

In this systematic review, we identified all mTORi conversion RCTs involving both sirolimus and everolimus with heterogeneous treatment and varying follow-up period. We included the RCTs with more than 20 cases in each group, which made the evidence in this review stronger and more convincing. We also evaluated some outcomes (graft function, acute rejection, graft loss, death) and some secondary outcomes that are not commonly reported in other systematic reviews such as drug discontinuation and adverse events. Consequently, the current study provides critical information to guide clinical decision-making on the mTORi conversion strategy after kidney transplantation.

Nevertheless, there were potential caveats limiting the generalizability of our findings. First, besides mTORi-induced AEs, the open-label nature of the 29 RCTs may have contributed to the high discontinuation rate in the conversion group. KTRs in the conversion group underwent a major change in immunosuppression after successful transplantation, and the KTRs may have tended to revert to their original immunosuppression due to the perceived limited efficacy or newly emergent AEs. Second, the 29 RCTs differed with respect to the baseline graft function, induction therapy, conversion time after transplantation, indication for conversion, strategy for conversion (abrupt vs. stepwise), and target immunosuppressant exposure. There may be selection and measurement bias, either of which may ultimately affect the validity of the study results. Third, all KTRs received mycophenolic acid (MPA)-based immunosuppression. It has been documented that CNIs, but not mTORi, inhibit MPA enterohepatic recirculation, resulting in a 50% lower MPA exposure (55). In most of the eligible RCTs, MPA was administered at a fixed dose; after conversion, more KTRs in the conversion group may have been overexposed to MPA, ultimately contributing to the reported AEs. Fourth, most RCTs focused on KTRs with low-to-moderate immunological risk; therefore, our results cannot be extended to high-risk KTRs. Fifth, due to the difference in the number of included cases, we did not distinguish the difference between CsA and TAC conversion.

Determining the optimal timing or indication for mTORi conversion requires a balance between avoiding the high rejection risk in the initial posttransplant period and minimizing the progressive development of CNI-related nephrotoxicity. Conversion ≥1-year posttransplant may be insufficient to prevent progression to graft dysfunction, except in KTRs with normal graft function and without proteinuria (14). Conversion in the first 6 months after transplantation seems appropriate for maintenance therapy in KTRs after careful screening (14). Refining selection criteria may help both in identifying patients who will profit most from switching and in alleviating the need to reintroduce CNI therapy. Primarily based on the current consensus, the target population for mTORi conversion should be KTRs with a baseline GFR of more than 40 ml/min and normal urinary protein excretion, an absence of previous AR and subclinical rejection, and appearance of donor-specific antibody (14, 25). Periodic surveillance biopsies may also help to identify a subset of KTRs who do not develop subclinical rejection or irreversible pathological changes in the renal interstitial to guide mTORi conversion therapy (56).

The high rate of drug discontinuation in the conversion group illustrates the challenge of handling a new spectrum of AEs that differs from more familiar CNI-related AEs, including dyslipidemia, acne, mouth ulceration, proteinuria, and AR (57, 58). Most AEs occurred early after conversion and in some cases resolved without intervention within a few weeks. Some of the discontinuations in the conversion group could have been avoided with better patient selection and more refined management of mTORi-related AEs. Specific patient monitoring and the concomitant use of lipid-lowering agents, angiotensin-converting-enzyme inhibitors, angiotensin receptor blockers, and induction therapies should be considered for combating dyslipidemia, proteinuria, and AR (16, 59). The identification of the optimal timing of conversion, appropriate candidates, dosing regimens for conversion, and safe management of mTORi-related AEs will be key in minimizing the need for discontinuation, which currently limits the broad applicability of this strategy.

## Data Availability Statement

The original contributions presented in the study are included in the article/**Supplementary Material**. Further inquiries can be directed to the corresponding authors.

## Author Contributions

JZ designed the study and wrote the manuscript. XF, QZ, and LL performed the meta-analysis and did the data extraction. SF, YF, TS, and ZH checked the results. XW and TL designed the research strategy and revised the manuscript. All authors reviewed the manuscript. All authors contributed to the article and approved the submitted version.

## Funding

This study was supported by grants from the Sichuan Science and Technology Program [grant number 2019YJ0133]; Chengdu Science and Technology Program [grant number 2019-YF05-00084-SN]; and 1.3.5 Project for Disciplines of Excellence-Clinical Research Incubation Project, West China Hospital, Sichuan University [grant number 2019-075, ZY2016104, 2021HXFH007].

## Conflict of Interest

The authors declare that the research was conducted in the absence of any commercial or financial relationships that could be construed as a potential conflict of interest.

## Publisher’s Note

All claims expressed in this article are solely those of the authors and do not necessarily represent those of their affiliated organizations, or those of the publisher, the editors and the reviewers. Any product that may be evaluated in this article, or claim that may be made by its manufacturer, is not guaranteed or endorsed by the publisher.

## Abbreviations

AE, adverse event; AR, acute rejection; ATG, anti-thymocyte globulin; CI, confidence interval; CNI, calcineurin inhibitor; CsA, cyclosporine A; DGF, delayed graft function; eGFR, estimated glomerular filtration rate; EVL, everolimus; IF/TA, interstitial fibrosis/tubular atrophy; KTR, kidney transplant recipient; MPA, mycophenolic acid; mTORi, mammalian target of rapamycin inhibitor; MD, mean difference; PTLD, posttransplant lymphoproliferative disease; RCT, randomized controlled trial; RR, risk ratio; SMD, standard mean difference; SRL, sirolimus; TAC, tacrolimus.
