# Supplementary material for: Conversion From Calcineurin Inhibitors to Mammalian Target of Rapamycin Inhibitors in Kidney Transplant Recipients: A Systematic Review and Meta-Analysis of Randomized Controlled Trials
Source: Front Immunol. 2021 Sep 3;12:663602. doi: 10.3389/fimmu.2021.663602 (PMC8446650; doi:10.3389/fimmu.2021.663602)
Supplement: Supplementary file 1 [file DataSheet_1.docx]

**Table S1**. Search strategy.

| Set Number | Search concept | Search string | Results |
| --- | --- | --- | --- |
| 1 | CNI | Tacrolimus or cyclosporine or calcineurin inhibitor or CNI | 46,368 |
| 2 | mTORi | Mammalian Target of Rapamycin Inhibitors or sirolimus or everolimus  or mTORi | 14,200 |
| 3 | Kidney transplantation | renal transplantation or kidney transplantation or kidney transplant or renal transplant or renal allograft or kidney allograft | 81,947 |
| 4 | Combine sets | 1 and 2 and 3 | 1,488 |

CNI, calcineurin inhibitor; mTORi, mammalian target of rapamycin inhibitor.

**Table S2.** Characteristics of the included studies.

| **Study ID** | **Study design** | **Study subject** | **Time of conversion** | **Graft function at time of randomization** | **Immunosuppressive regimen** | | | **Target drug levels** | | | **Outcomes reported (Intervention vs. Comparator)** | | | | | **Follow-up**  **(month)** |
| --- | --- | --- | --- | --- | --- | --- | --- | --- | --- | --- | --- | --- | --- | --- | --- | --- |
|  |  |  |  |  |  |  |  | **Target CNI levels (ng/ml)** | | **Target mTORi levels (ng/ml)** | **eGFR**  **mean (SD)**  **mL/min/1.73 m^2^** | **sCr**  **umol/L** | **Mortality**  **n (%)** | **Graft loss**  **n (%)** | **Rejection**  **n (%)** |  |
|  |  |  |  |  | **Pre-conversion** | **Intervention** | **Comparator** | **Before randomization** | **After randomization** |  |  |  |  |  |  |  |
| Tönshoff 2019 | RCT (N=106) | pediatric patients | 4 to 6 weeks  posttransplant | Stable function:  eGFR >40 mL/min/1.73m^2^ | CNI + MMF + steroids | EVL  (N=52) | TAC  (N=54) | TAC:  8 - 12 | TAC:  2 - 4 | EVL:  3 ‑ 8 | 76.2 vs. 72.5 | NR | 0 vs. 0 | 0 vs. 0 | BPAR  7 (13.5%)  vs.  1 (1.9%) | 12 |
| Taber  2019 | RCT  (N=60) | adult patients | 3 months  posttransplant | Stable function:  eGFR >30 mL/min/1.73m^2^ | CNI +  MPA + steroids | EVL  (N=30) | TAC  (N=30) | TAC:  8 - 12 | TAC:  5 - 8 | EVL:  3 ‑ 8 | 59 (14)  vs.  56 (15) | NR | 0 vs. 0 | 0 vs. 0 | AR  1 (3%)  vs.  2 (7%) | 12 |
| Brakemeier  2019 | RCT  (N=75) | senior  patients | 7 weeks  posttransplant | Stable function:  sCr<3.0 mg/dL,  proteinuria <500 mg/g | CNI +  MPA + steroids | EVL  (N=51) | CsA  (N=24) | CsA:  100 - 150 | CsA:  111.0 ± 33.0 | EVL:  5 - 10 | 36.5 (10.8)  vs.  42.0 (13.0) | 158.3  vs.  162.8 | 0 vs. 0 | 0 vs. 0 | BPAR  12 (29.2%)  vs.  5 (20.8%) | 6 |
| Haynes  2018 | RCT  (N=394) | adult patients | 6 months posttransplant | Stable function:  5-7 months after transplantation | CNI +  MPA + steroids | SRL  (N=197) | TAC  (N=197) | NR | TAC:  5 - 7 | SRL:  5 - 10 | 53.5 (16.8)  vs.  52.6 (16.6) | NR | 11 (5.6%)  vs.  9 (4.6%) | 8 (4.1%)  vs.  4 (2.0%) | BPAR  29 (14.7%)  vs.  6 (3.0%) | 18 |
| Bouamar  2018 | RCT  (N=60) | adult patients | 3 months posttransplant | NR | CNI +  MMF + steroids | EVL  (N=30) | TAC  (N=30) | NR | TAC:  5 - 10 | EVL:  4 - 7 | 53 (13)  vs.  56 (18) | 108  vs.  121 | 0 (0%)  vs.  1 (3.3%) | 1 (3.3%) vs.  0 (0%) | BPAR  9 (30.0%) vs. 2 (6.7%) | 12 |
| Fijter  2017 | RCT  (N=715) | adult patients | 10 - 14 weeks posttransplant | sCr <250 umol/L and eGFR≥25 mL/min/1.73m^2^ | CNI +  MPS + steroids | EVL  (N=359) | CNI  (N=356) | TAC:  C_0_ 6 - 12  CsA:  150 - 300 | TAC:  C_0_ 5 - 10  CsA: 100 - 250 | EVL:  6 - 10 | 62.5 (22.4)  vs.  57.4 (19.9) | NR | 8 (2.6%)  vs.  9 (2.6%) | 4 (1.2%)  vs.  4 (1.1%) | BPAR  39 (10.9%)  vs.  21 (5.9%) | 24 |
| Budde  2017 | RCT  (N=336) | adult patients | 3 months posttransplant | sCr < 3.0 mg/dL | CNI +  MPS + steroids | EVL  (N=171) | CsA  (N=165) | CsA:  150 - 220 | CsA:  150 - 220 | EVL:  3 - 8 | 71.7 (19.4)  vs.  62.5 (17.0) | NR | 2 (1.2%)  vs.  3 (1.8%) | 2 (1.2%) vs.  1 (0.6%) | BPAR  20 (11.7%)  vs.  13 (7.9%) | 12 |
| Bemelman  2017 | RCT  (N=336) | adult patients | 6 months posttransplant | No rejection in the 6-month | CNI +  MPS + steroids | EVL  (N=96) | CsA  (N=89) | CsA:  AUC_12_: 3250 ug*h/L | CsA:  AUC_12_: 3250 ug*h/L | EVL:  AUC_12:_150 ug*h/L | NR | NR | 4 (4.2%)  vs.  1 (1.1%) | 0  vs.  3 (3.4%) | AR  12 (13%)  vs.  17 (19%) | 24 |
| Pretagostini  2016 | RCT  (N=49) | adult patients | 1-month posttransplant | NR | CNI +  MPS + steroids | EVL  (N=24) | TAC  (N=25) | TAC:  6 - 12 | TAC:  4 - 6 | EVL:  5 - 10 | NR | 123.8 vs.  159.1 | NR | NR | AR  2 (8.3%) vs.  3 (12.0%) | 12 |
| Felix  2016 | RCT  (N=119) | adult patients | 3 months posttransplant | eGFR > 40 ml/min, proteinuria < 0.5g/day | CNI +  MPS + steroids | SRL  (N=60) | TAC  (N=59) | TAC:  8 - 15 | TAC:  5 - 15 | SRL  5 - 15 | NR | NR | 1 (2.7%)  vs.  1 (2.8%) | NR | BPAR  9 (15.0%)  vs.  2 (3.4%) | 24 |
| Cruzado  2016 | RCT  (N=71) | adult patients | 6 months - 3 years  posttransplant | eGFR > 40 ml/min,  proteinuria < 0.5g/day | CNI +  MPA+ steroids | EVL  (N=35) | TAC  (N=36) | TAC:  7.2 ± 2.0 | TAC:  7.0 ± 2.3 | EVL:  5 - 8 | 60.8  vs.  57.7 | NR | 1 (2.9%)  vs.  0 (0.0%) | 0 vs. 0 | 0 vs. 0 | 24 |
| Rostaing  2015 | RCT  (N=194) | adult patients | 3 – 4 months  posttransplant | month 3 biopsy with a function graft | CNI +  MPS + steroids | EVL  (N=96) | CsA  (N=98) | CsA:  1100 - 1300 | CsA:  400 - 600 | EVL:  6 - 10 | 52.1 (15.9)  vs.  50.2 (15.3) | NR | 0 vs. 0 | 5 (5.2%) vs.  1 (1.0%) | BPAR  24 (25.0%) vs. 5 (5.1%) | 12 |
| Mjörnstedt  2015 | RCT  (N=182) | adult patients | 7 weeks  posttransplant | Stable function:  No rejection | CNI +  MPS + steroids | EVL  (N=92) | CsA  (N=90) | CsA:  C_0_ 75 - 200 | CsA:  C_0_ 50 - 150 | EVL:  6 - 10 | 60.1 (19.7)  vs.  57.4 (20.2) | 128  vs.  140 | 1 (1.1%)  vs.  3 (3.3%) | 0 vs. 0 | BPAR  12 (13.0%) vs.  10 (11.1%) | 24 |
| Sandes-Freitas 2015 | RCT  (N=93) | adult patients | 3 months posttransplant | NR | CNI +  MPS + steroids | SRL  (N=48) | TAC  (N=45) | TAC:  8 - 10 | TAC:  5 - 7 | SRL:  8 - 12 | 63.7 (19.3)  vs.  68.1 (14.9) | 114.9 vs. 106.1 | NR | NR | BPAR  4 (7.3%)  vs. 0 | 24 |
| Budde  2015 | RCT  (N=93) | adult patients | NR | stable graft function sCr< 221 µmol/L | CNI +  MPS + steroids | EVL  (N=46) | CNI  (N=47) | TAC: 5 - 10  CsA: 80 - 150 | TAC: 5 - 10  CsA: 80 - 150 | EVL:  6 - 10 | 63.8 (19.8)  vs.  58.2(16.6) | NR | 1 (2.2%)  vs.  1 (2.1%) | 0 vs. 0 | BPAR  0 vs. 0 | 12 |
| Silva  2013 | RCT  (N=204) | adult patients | 3 months posttransplant | Stable graft function | CNI+  MPS + steroids | SRL  (N=97) | TAC  (N=107) | TAC:  C_0_: 10 - 15 | TAC:  C_0_: 10 - 15 | SRL:  8 - 12 | 66.2 (25.3)  vs.  70.7 (25.1) | 123.8 vs. 114.9 | 3 (3.1%)  vs.  3 (2.8%) | 1 (1.0%) vs.  1 (0.9%) | BPAR  28 (28.9%) vs.  24 (22.4%) | 24 |
| Chhabra  2013 | RCT  (N=187) | adult patients | 12 months posttransplant | NR | CNI +  MMF + steroids | SRL  (N=123) | TAC  (N=64) | TAC:  8 - 10 | TAC:  6 - 8 | SRL:  5 - 8 | 66.5 (23.7)  vs.  66.0 (18.2) | NR | 4 (3.3%)  vs.  0 | 3 (2.4%) vs.  2 (3.1%) | BPAR  12 (9.8%)  vs.  6 (9.5%) | 24 |
| Bansal  2013 | RCT  (N=60) | adult patients | 2 months posttransplant | sCr< 1.2 mg/dl, proteinuria < 500 mg/day. | CNI +  MMF + steroids | SRL  (N=31) | CNI  (N=29) | TAC: 8 - 10  CsA: 200 - 300 | TAC: 6 - 8  CsA: 150 - 250 | SRL:  8 - 15 | 88.9 (11.8)  vs.  80.6 (16.5) | 87.5  vs. 100.8 | NR | NR | AR  0 vs.  2 (6.9%) | 6 |
| Mjörnstedt  2012 | RCT  (N=102) | adult patients | 7 weeks posttransplant | Rejection-free | CNI +  MPS + steroids | EVL  (N=102) | CNI  (N=100) | CsA:  75 - 200 | CsA:  50 - 150 | EVL:  6 - 10 | 73.9 (17.1)  vs.  71.3 (17.6) | 122  vs.  132 | 2 (2.0%)  vs.  2 (2.0%) | 0 vs. 0 | BPAR  28 (27.5%) vs.  11 (11.0%) | 12 |
| Weir  2011 | RCT  (N=299) | adult patients | 1-6 months  posttransplant | No rejection, eCrCl >30 mL/min, sCr <2.5 mg/dL | CNI +  MMF + steroids | SRL  (N=148) | CNI  (N=151) | CNI: NR | NR | SRL:  5 - 10 | 75.5 (19.2)  vs.  71.2 (23.5) | 127.1 vs. 151.8 | 0  vs.  5 (3.3%) | 3 (2.0%) vs. 6 (4.0%) | BPAR  14 (9.5%)  vs.  17 (11.3%) | 24 |
| Holdaas  2011 | RCT  (N=250) | adult patients | 6 months posttransplant (mean time 5.4 years posttransplant) | Renal impairment, GFR of 30 - 70 mL/min | CNI +  MPA+ steroids | EVL  (N=127) | CNI  (N=123) | CsA：  C_2_: ≥400  TAC：  C_0_: ≥4 | CsA：  C_2_: ≥400  TAC：  C_0_: ≥4 | EVL：  8 - 12 | 48.0 (22.0)  vs.  46.0 (20.4) | 171  vs.  168 | 3 (2.4%)  vs.  0 | 4 (3.1%) vs.  6 (4.9%) | BPAR  7 (5.5%)  vs.  3 (2.4%) | 24 |
| Heilman  2011 | RCT  (N=122) | adult patients | 1-month posttransplant | No BPAR | CNI +  MMF+ steroids | SRL  (N=62) | TAC  (N=60) | TAC:  10 - 12 | TAC:  5 – 8 | SRL:  8 | 64.3 (29.0)  vs.  62.8 (21.6) | 122.9 vs. 111.4 | 1 (1.6%)  vs.  2 (3.3%) | 1 (1.6%) vs.  2 (3.3%) | 8 (13%)  vs.  1 (2%) | 24 |
| Budde  2011 | RCT  (N=300) | adult patients | 4.5 months  posttransplant | No rejection, sCr <265.2 µmol/mL | CNI +  MPS + prednisone | EVL  (N=154) | CsA  (N=146) | CsA:  C_0_: 150 - 220  C_2_: 1100 - 1400 | CsA:  C_0_: 100 - 150  C_2_: 500 - 800 | EVL：  6 - 10 | 71·8 (18)  vs.  61·9 (18) | 141.7  vs.  137.7 | 0  vs.  1 (<1%) | 0 vs. 0 | BPAR  23 (15%)  vs.  22 (15%) | 12 |
| Guba  2010 | RCT  (N=140) | adult patients | 10-24 days  posttransplant | Low-to-moderate immunological risk,  no rejection | CNI +  MMF + prednisone | SRL  (N=69) | CsA  (N=71) | CsA:  C_0_: 200 - 250 | CsA:  C_0_:100 - 150 | SRL：  5 - 10 | 64.5 (25.2)  vs.  53.4 (18.0) | 133.5 vs. 165.3 | 1 (1.4%)  vs.  1 (1.4%) | 1 (1.4%) vs.  3 (4.2%) | BPAR  12 (17.4%) vs.  11 (15.5%) | 12 |
| Schena  2009 | RCT  (N=830) | adult patients | 6-120 months  posttransplant | Functioning allograft, eGFR ≥20 mL/min | CNI +  MMF + prednisone | SRL  (N=555) | CNI  (N=275) | NR | CsA:  C_0_: 50 – 250  TAC:  C_0_: 4 - 10 | SRL：  C0: 8 - 20 | 59 (15.4)  vs.  57.7 (15.4) | NR | NR | 27 (4.9%) vs.  8 (2.9%) | BPAR  44 (7.9%)  vs.  19 (6.9%) | 24 |
| Lebranchu  2009 | RCT  (N=192) | adult patients | 3 months  posttransplant | eGFR >40 mL/min, proteinuria < 1g/24h | CNI +  MMF + prednisone | SRL  (N=95) | CsA  (N=97) | CsA:  800 -1200 | CsA:  500‑800 | SRL:  5-10 | 61.2 (14.6)  vs.  53.9 (7) | 117.4 vs. 132.3 | 0 vs. 0 | 1 (1.1%) vs.  0 | BPAR  16 (16.8%) vs.  8(8.2%) | 12 |
| Bemelman  2009 | RCT  (N=77) | adult patients | 6 months  posttransplant | No rejection | CNI +  MPA + prednisone | EVL  (N=38) | CsA  (N=39) | CsA :  AUC_12_ 3250 ug*h/L | NR | EVL：  AUC12 150 ug*h/L | 55 (20)  vs.  44 (15) | NR | NR | NR | BPAR  0 vs.  1 (2.6%) | 24 |
| Liu  2007 | RCT  (N=110) | adult patients | More than 12 months  posttransplant | No rejection | CNI +  MMF + prednisone | SRL  (N=56) | CsA  (N=54) | NR | CsA:  C_2_: 400 - 500 | SRL:  C0: 5 - 10 | NR | 150.4 vs. 210.2 | NR | NR | NR | 24 |
| Stallone  2005 | RCT  (N=84) | adult patients | 12-36 months  posttransplant | Biopsy-proven CAN | CNI +  MMF + prednisone | SRL  (N=34) | CNI  (N=50) | NR | CsA:  C_2_: 400-500  Tac: C_0_: 4 - 6 | SRL:  C_0_: 6 - 10 | NR | 164.4 vs. 175.9 | NR | NR | AR  0 vs. 0 | 24 |

CNI, calcineurin inhibitor; mTORi, mammalian target of rapamycin inhibitor; eGFR, estimated glomerular filtration rate; sCr, serum creatinine; RCT, randomized controlled trial; MMF, mycophenolate mofetil; EVL, everolimus; TAC, tacrolimus; NR, not reported; BPAR, biopsy-proven acute rejection; MPA, mycophenolic acid; AR, acute rejection; CsA, cyclosporine A; SRL, sirolimus; AUC, area under curve; MPS, mycophenolate sodium; eCrCl, estimated creatinine clearance rate; CAN, chronic allograft nephropathy.

**Table S3**. Meta-analysis results of conversion to mTORi vs. CNI continuation for primary and secondary endpoints

| Outcomes | RCTs | RR(MD) | Heterogeneity | |
| --- | --- | --- | --- | --- |
|  | (n) | [95%CI] | p | I^2^ |
| **Primary endpoints** | | | | |
| eGFR  (mL/min/1.73m^2^) | 22 | 0.20 [0.10, 0.31] | <0.01 | 66% |
| Graft loss (n) | 22 | 1.14 [0.75, 1.74] | 0.64 | 0 |
| BPAR (n) | 28 | 1.58 [1.22, 2.04] | <0.01 | 50% |
| Mortality (n) | 22 | 0.99 [0.64, 1.55] | 0.87 | 0 |
| **Secondary endpoints** | | | | |
| Any adverse events (n) | 15 | 1.03 [0.99, 1.06] | <0.01 | 70% |
| Any serious adverse events (n) | 16 | 1.23 [1.09, 1.39] | <0.01 | 54% |
| Discontinue of drugs (n) | 21 | 2.52 [1.75, 3.63] | <0.01 | 78% |
| Any infections (n) | 16 | 1.15 [1.01, 1.31] | <0.01 | 74% |
| Any serious infections (n) | 8 | 1.17 [1.05, 1.30] | 0.43 | 0 |
| CMV infection (n) | 16 | 0.78 [0.50, 1.22] | <0.01 | 64% |
| BKV infection (n) | 13 | 0.76 [0.39, 1.49] | 0.06 | 43% |
| Proteinuria (n) | 18 | 1.87 [1.34, 2.59] | <0.01 | 59% |
| Malignancy (n) | 15 | 0.74 [0.55, 0.99] | 0.36 | 8% |
| Increased sCr (n) | 8 | 0.77 [0.58, 1.02] | 0.54 | 0 |
| IF/TA (n) | 6 | 0.92 [0.66, 1.29] | 0.02 | 67% |
| Diabetes (n) | 16 | 1.17 [0.89, 1.55] | 0.77 | 0 |
| Ulcer (n) | 8 | 11.70 [6.18, 22.17] | 0.98 | 0 |
| Anemia (n) | 12 | 1.55 [1.26,1.89] | 0.07 | 41% |
| Leukopenia (n) | 11 | 1.56 [1.27,1.91] | 0.10 | 38% |
| Thrombocytopenia (n) | 3 | 2.45 [1.13,5.35] | 0.28 | 21% |
| Dyslipidemia (n) | 11 | 1.41 [1.27,1.58] | <0.01 | 63% |
| Diarrhea (n) | 15 | 1.46 [1.28,1.67] | 0.02 | 49% |
| Gastroenteritis (n) | 5 | 0.98 [0.70,1.37] | 0.63 | 0 |
| Edema (n) | 6 | 1.49 [1.14,1.93] | 0.20 | 31% |
| Acne (n) | 7 | 6.43 [3.43,12.04] | 0.89 | 0 |
| Wound-related problem (n) | 3 | 1.13 [0.82,1.56] | 0.46 | 0 |
| PTLD (n) |  | 3.10 [0.33,29.20] | 1.00 | 0 |
| Pyrexia (n) | 3 | 1.04 [0.50,2.17] | 0.54 | 0 |

RCT, randomized controlled trial; RR, risk ratio; MD, mean difference; CI, confidence interval; eGFR, estimated glomerular filtration rate; BPAR, biopsy-proven acute rejection; CMV, cytomegalovirus; BKV, BK virus; sCr, serum creatinine; IF/TA, interstitial fibrosis / tubular atrophy; PTLD, posttransplant lymphoproliferative disease.
