# Supplementary material for: Conversion From Calcineurin Inhibitors to Mammalian Target of Rapamycin Inhibitors in Kidney Transplant Recipients: A Systematic Review and Meta-Analysis of Randomized Controlled Trials
Source: Front Immunol. 2021 Sep 3;12:663602. doi: 10.3389/fimmu.2021.663602 (PMC8446650; doi:10.3389/fimmu.2021.663602)
Supplement: Supplementary file 2 [file DataSheet_2.docx]

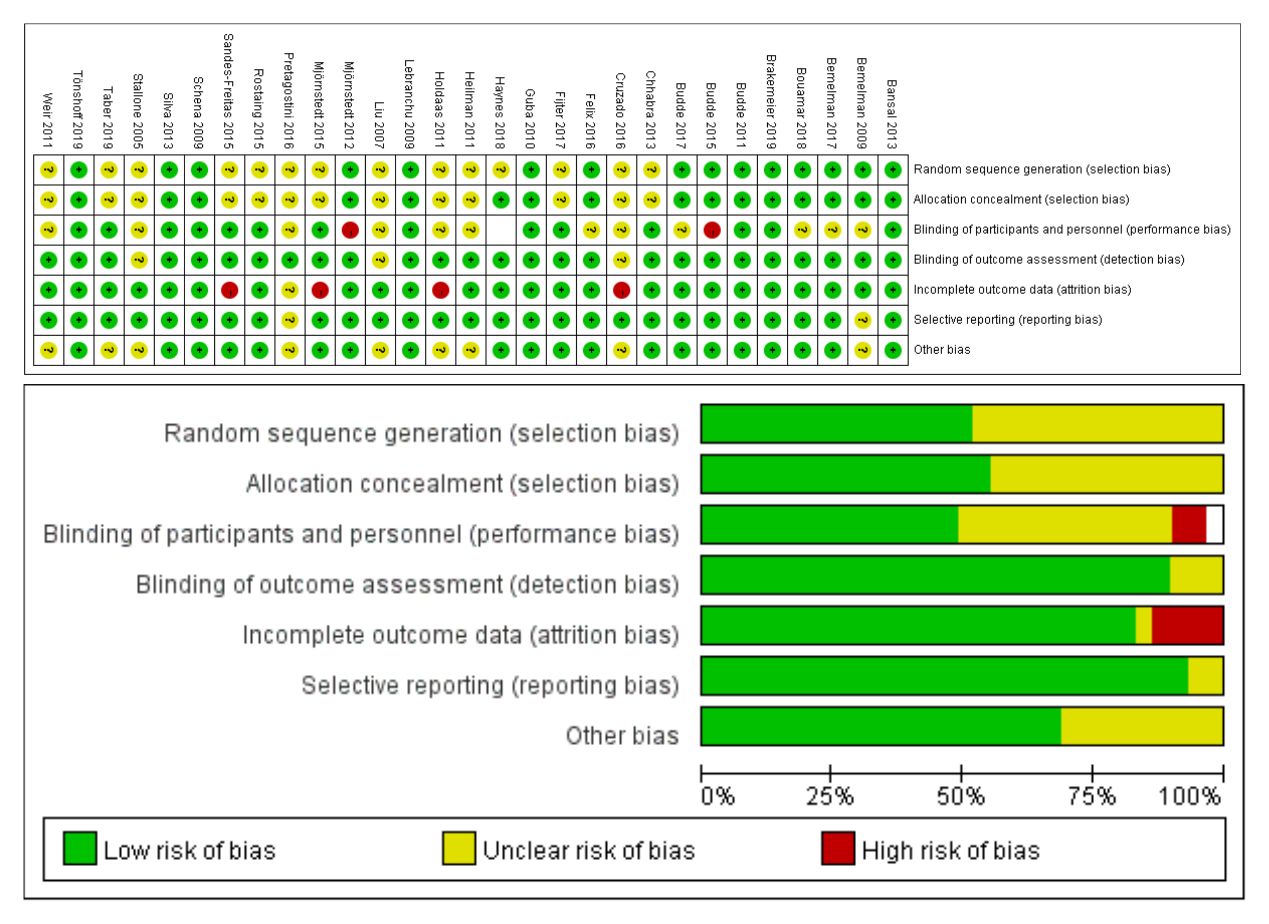


**Figure S1**. Risk of bias assessment.


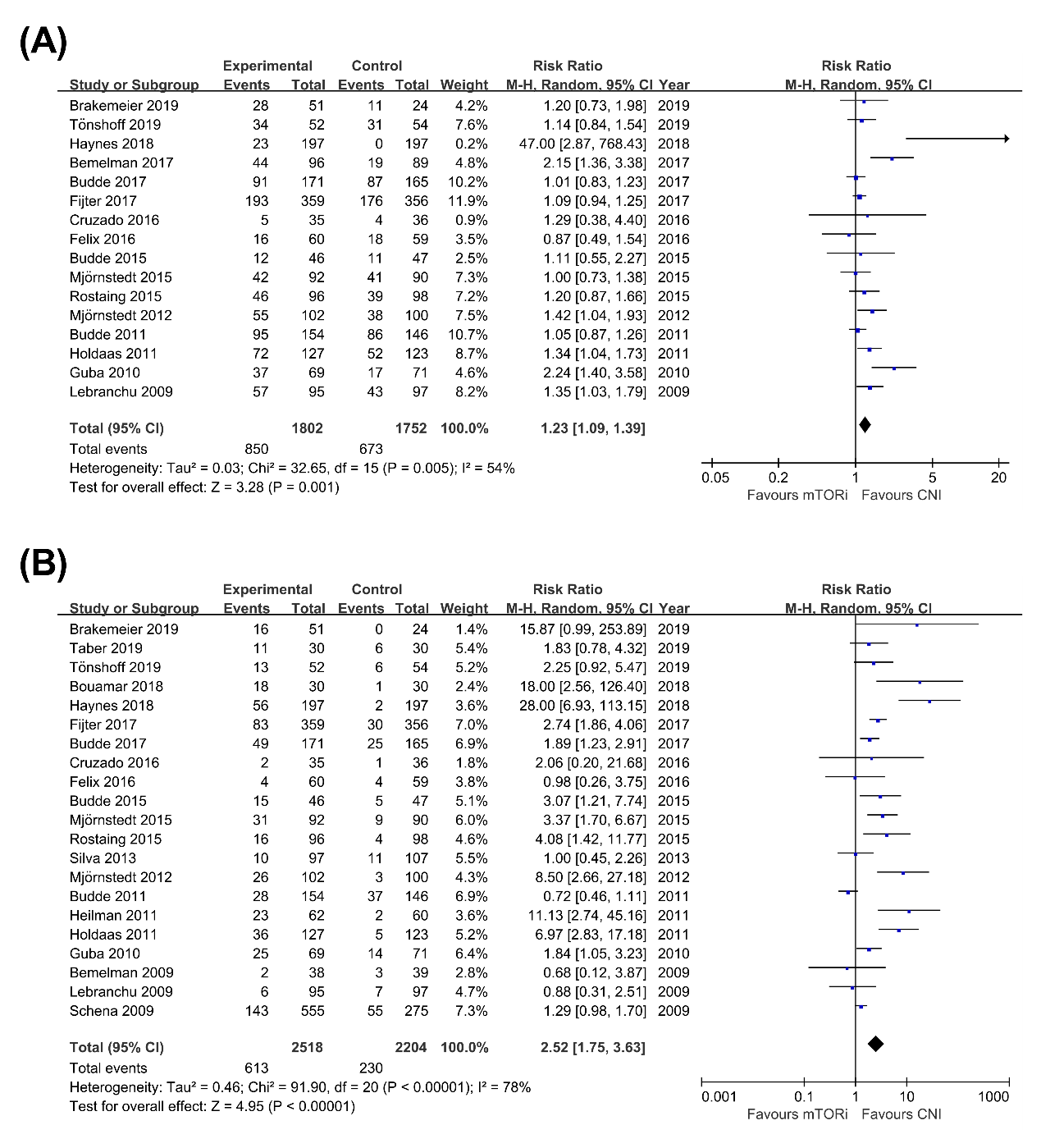


**Figure S2**. The forest plot of studies that compared conversion from CNIs to mTOR inhibitors versus maintenance of CNI therapy for the outcomes of (A) Serious adverse events, (B) Drug discontinuation.


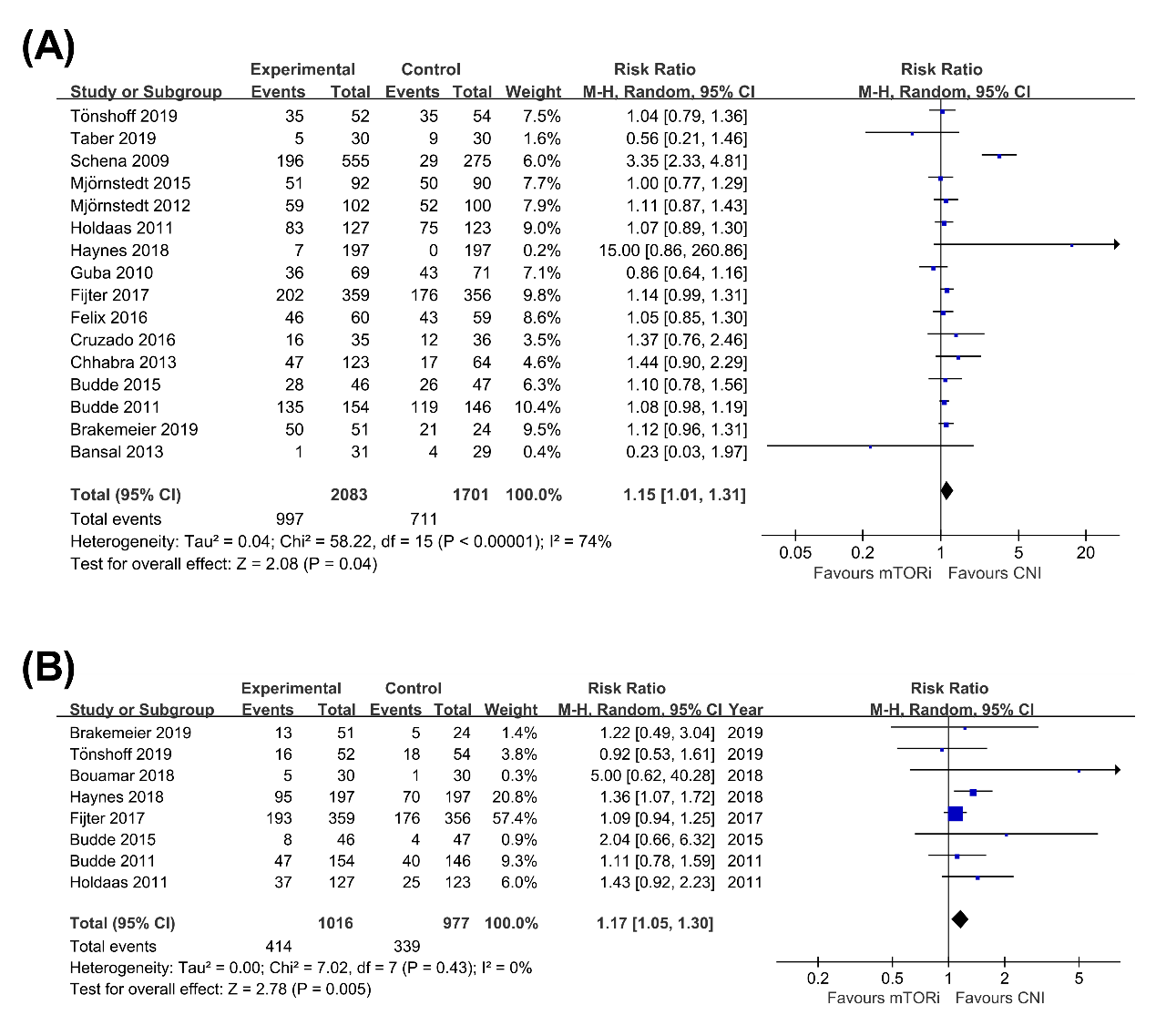


**Figure S3**. The forest plot of studies that compared conversion from CNIs to mTOR inhibitors versus maintenance of CNI therapy for the outcomes of (A) Infections, (B) Serious infections.


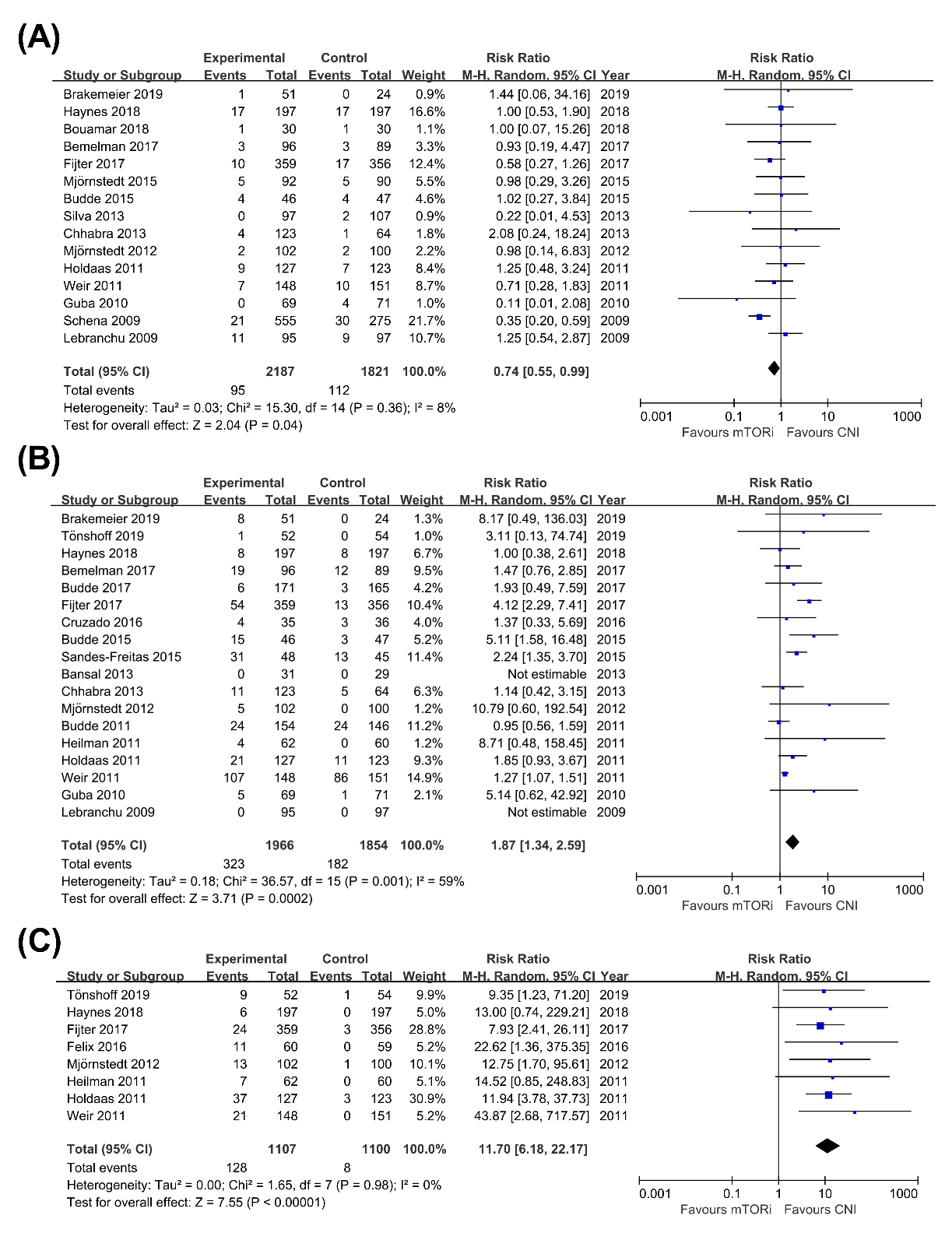


**Figure S4**. The forest plot of studies that compared conversion from CNIs to mTOR inhibitors versus maintenance of CNI therapy for the outcomes of (A) Malignancy, (B) Proteinuria, (C) Mouth Ulcer.


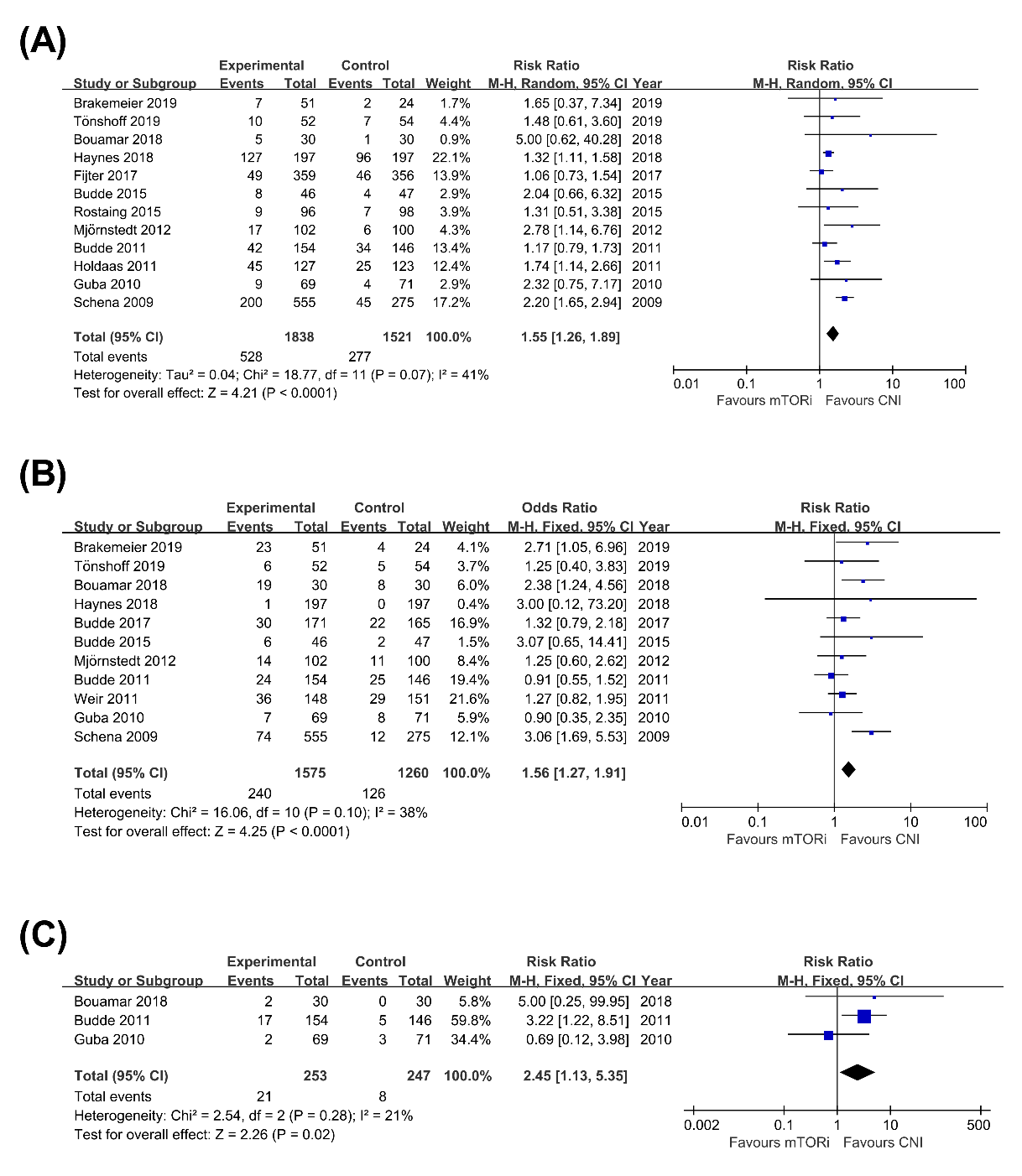


**Figure S5**. The forest plot of studies that compared conversion from CNIs to mTOR inhibitors versus maintenance of CNI therapy for the outcomes of (A) Anemia, (B) Leukopenia, (C) Thrombocytopenia.


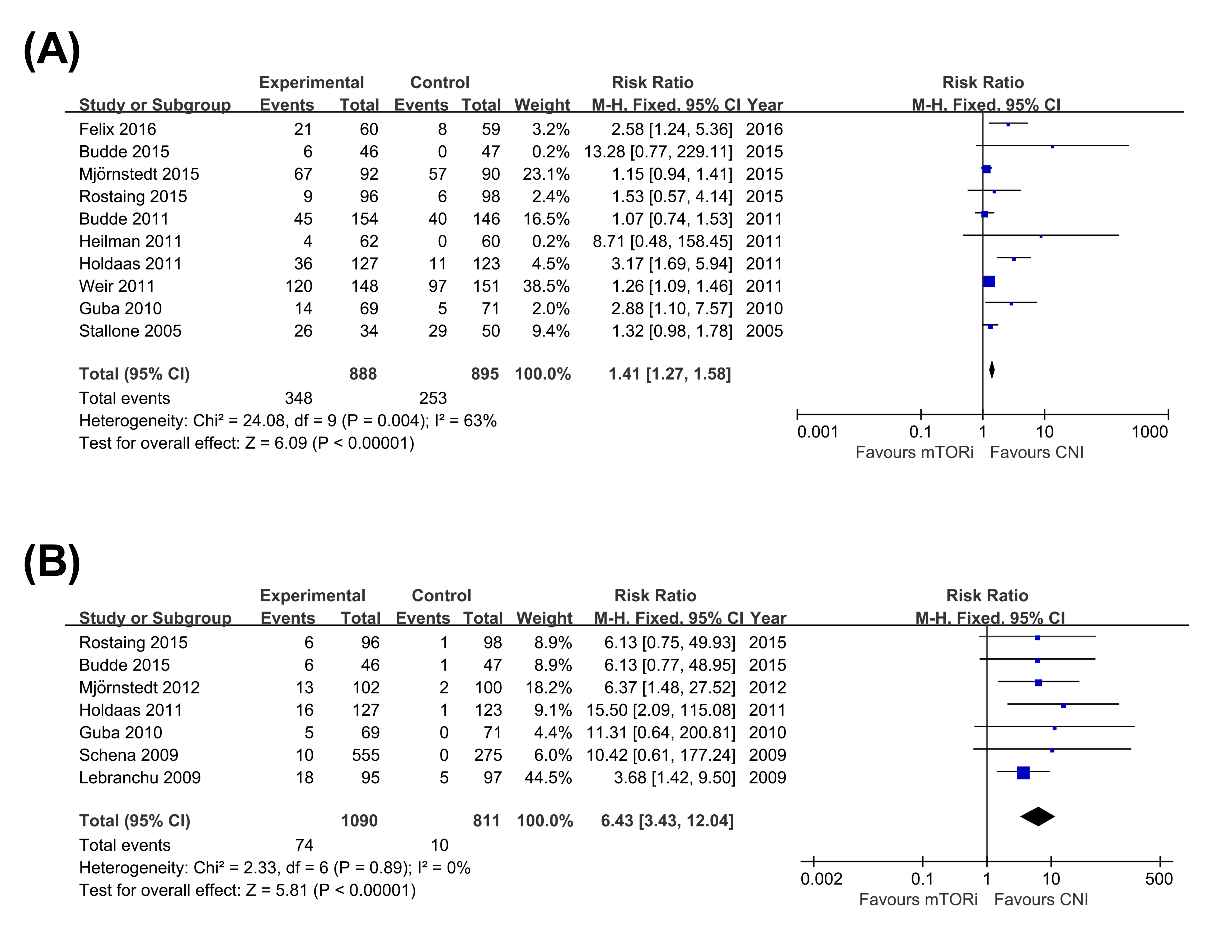


**Figure S6**. The forest plot of studies that compared conversion from CNIs to mTOR inhibitors versus maintenance of CNI therapy for the outcomes of (A) Dyslipidemia, (B) Acne.


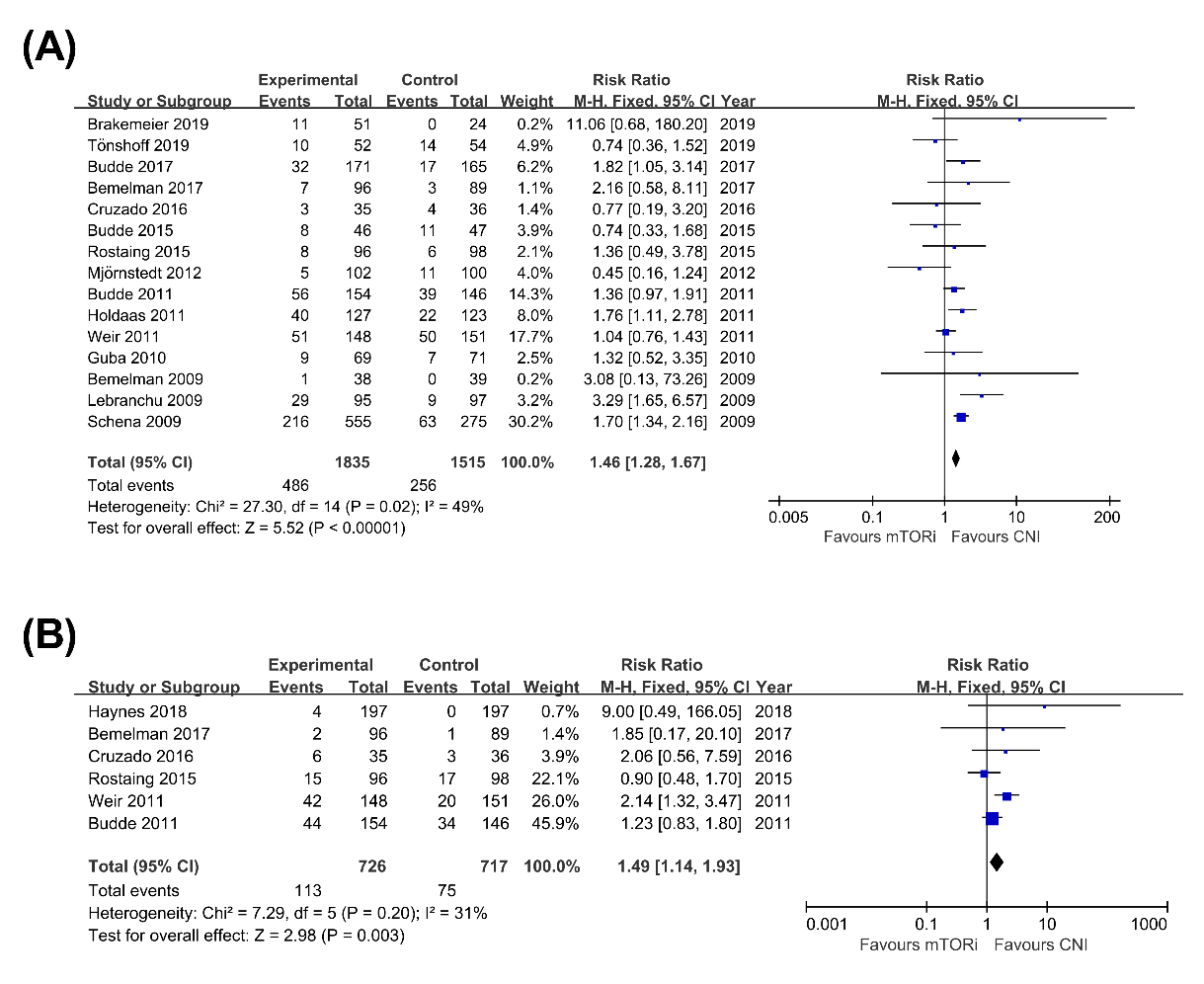


**Figure S7**. The forest plot of studies that compared conversion from CNIs to mTOR inhibitors versus maintenance of CNI therapy for the outcomes of (A) Diarrhea, (B) Edema.


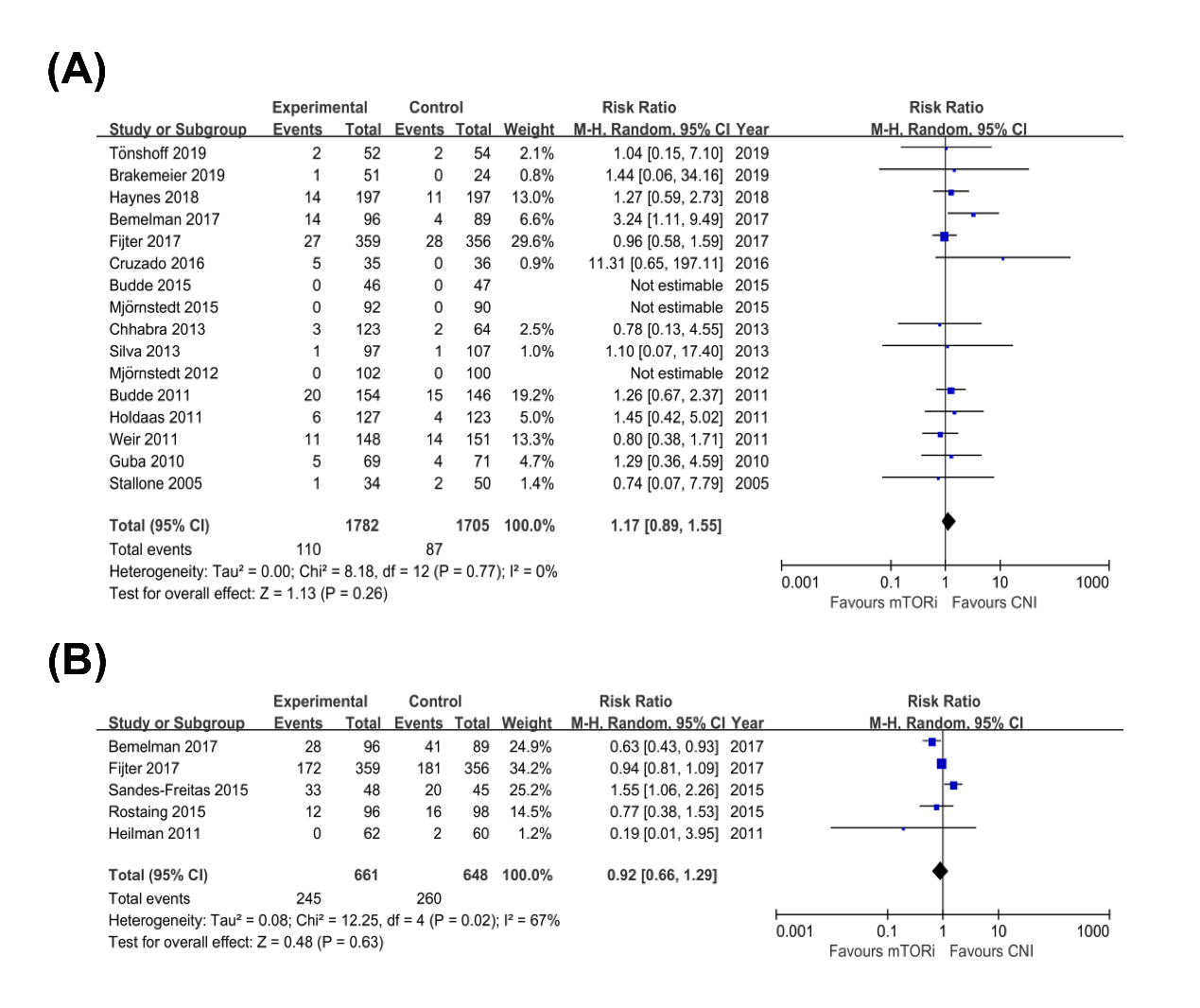


**Figure S8**. The forest plot of studies that compared conversion from CNIs to mTOR inhibitors versus maintenance of CNI therapy for the outcomes of (A) Diabetes, (B) IF/TA.


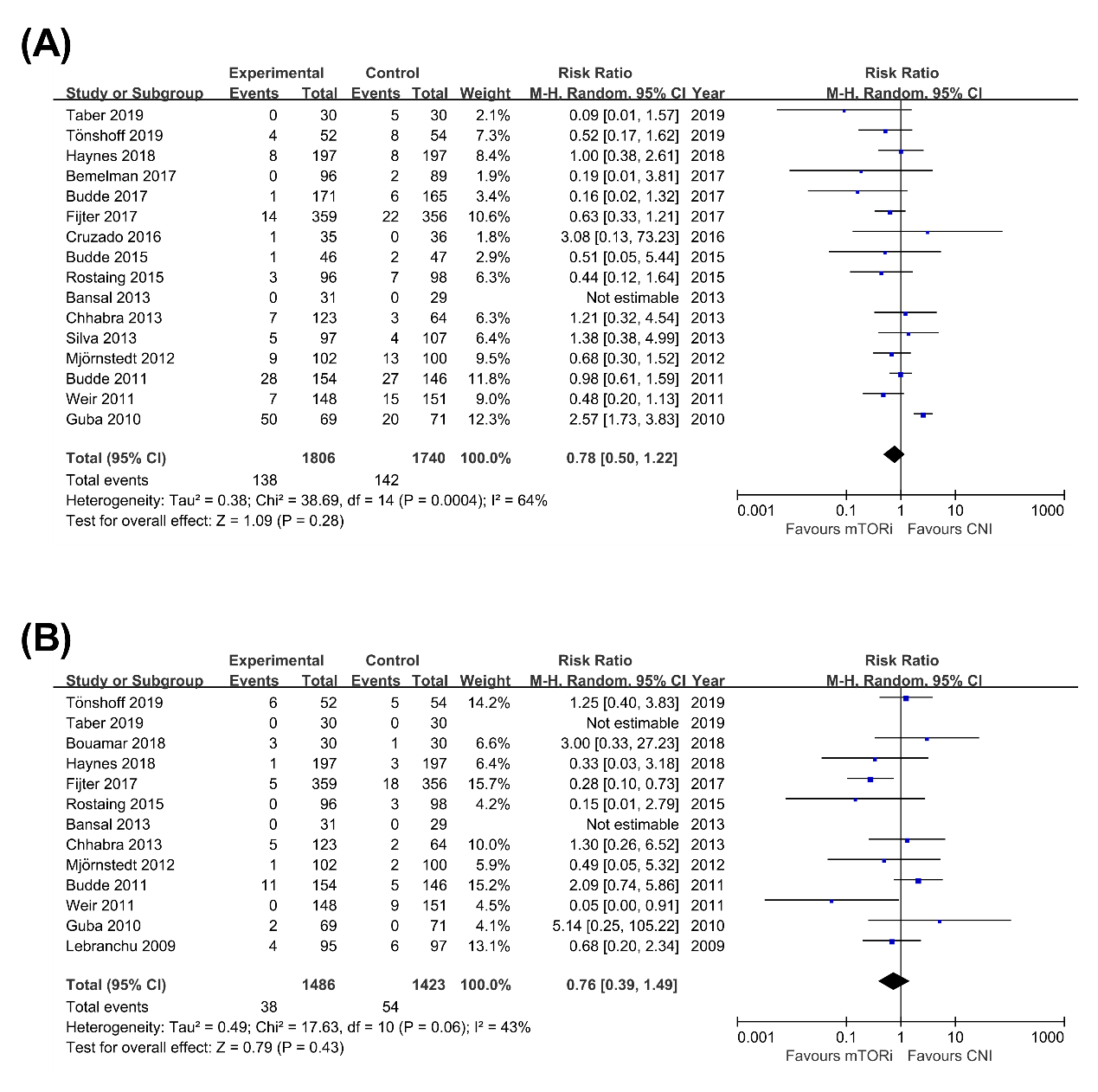


**Figure S9**. The forest plot of studies that compared conversion from CNIs to mTOR inhibitors versus maintenance of CNI therapy for the outcomes of (A) CMV infection, (B) BKV infection.
